# Jolfa Urban Safety audit based on Geographical Information System (GIS)

**Published:** 2019-08

**Authors:** Pedram Beiglo, Fatemeh Moniri Alamdari, Javad Kamusi Alamdari

**Affiliations:** ^*a*^Transportation Engineering and planning, Bogazici University, Turkey.; ^*b*^Geography and Urban Planning, Department of Geography, Astara Branch, Islamic Azad university, Astara, Iran.

## Abstract

**Background:**

Considering the importance of traffic accidents in cities and the role of non-standard urban roads in increasing them, it is important to detect and reform road safety defects. In order to implement Safe Community Program of Jolfa safe city, a method was introduced for detecting and reform managing of road safety defects.

**Methods:**

Urban roads of Jolfa were filmed in eleven separate routes for eight hours by Timestamp Camera software.Safety issues were identified and in eleven layers (Geometric design, sidewalks, lining, pavement, speed bumps, school districts, shopping malls entries, constructions waste, night vision, pedestrian crossings And signs) were entered in GIS.To prioritize reforming actions, the compressibilityof layers of each safety defects category were formed in order toidentify the concentration of each type of road safety defects. If the case of reforming, the operator places this point in modified category and upload the reformed image in GIS.The result of this method was clearly seen in work progress reports.

**Results:**

Overly 856 safety issues were identifies in 11 safety categories. Two main area was extracted as prioritized areas with the highest frequency of identified safety issues. The GIS map was presented in the traffic safety committee and action plan was developed for safety promotion interventions.

**Conclusions:**

Urban safety audit based on GIS provides a proper information base for city managers about the safety and facilitates the safety management rules for communities who implementing safe community program.

**Keywords:**

Safe Community, Jolfa city, Urban audit, GIS

